# Cortical Morphology Alterations Mediate the Relationship Between Glymphatic System Function and the Severity of Asthenopia

**DOI:** 10.1155/ijbi/4464776

**Published:** 2025-02-25

**Authors:** Yilei Chen, Jun Xu, Yingnan Kong, Yingjie Kang, Zhigang Gong, Hui Wang, Yanwen Huang, Songhua Zhan, Ying Yu, Xiaoli Lv, Wenli Tan

**Affiliations:** ^1^Department of Radiology, Shuguang Hospital Affiliated to Shanghai University of Traditional Chinese Medicine, Shanghai, China; ^2^Department of Pharmacy, Shanghai Municipal Hospital of Traditional Chinese Medicine, Shanghai University of Traditional Chinese Medicine, Shanghai, China; ^3^Department of Ophthalmology, Shuguang Hospital Affiliated to Shanghai University of Traditional Chinese Medicine, Shanghai, China; ^4^Department of Ophthalmology, The Second Affiliated Hospital of Zhejiang Chinese Medical University, Hangzhou, China

**Keywords:** asthenopia, diffusion tensor image analysis along the perivascular space, glymphatic system, surface-based morphometry

## Abstract

**Objectives**: This study is aimed at assessing glymphatic function by diffusion tensor image analysis along the perivascular space (DTI-ALPS) and its associations with cortical morphological changes and severity of accommodative asthenopia (AA).

**Methods**: We prospectively enrolled 50 patients with AA and 47 healthy controls (HCs). All participants underwent diffusion tensor imaging (DTI) and T1-weighted imaging and completed the asthenopia survey scale (ASS). Differences in brain morphometry and the analysis along the perivascular space (ALPS) index between the two groups were compared. The correlation and mediation analyses were conducted to explore the relationships between them.

**Results**: Compared to HCs, patients with AA exhibited significantly increased sulcal depth in the left superior occipital gyrus (SOG.L) and increased cortical thickness in the left superior temporal gyrus (STG.L), left middle occipital gyrus (MOG.L), left postcentral gyrus (PoCG.L), and left precuneus (PCUN.L). Additionally, patients with AA had a significantly lower ALPS index than HCs. The sulcal depth of the SOG.L was significantly positively correlated with the ASS score in patients with AA, and a positive correlation was found between the cortical thickness of the MOG.L and ASS score. The ALPS index was negatively associated with the sulcal depth of the SOG.L and cortical thickness of the MOG.L. Mediation analysis revealed that the sulcal depth of SOG.L and cortical thickness of MOG.L partially mediated the impact of the DTI-ALPS index on the ASS score.

**Conclusion**: Our findings suggested that patients with AA exhibit impaired glymphatic function, which may contribute to the severity of asthenopia through its influence on cortical morphological changes. The ALPS index is anticipated to become a potential imaging biomarker for patients with AA.

**Trial Registration:** Chinese Registry of Clinical Trials: ChiCTR1900028306

## 1. Introduction

Due to the prolonged overuse of digital devices such as computers, mobiles, and tablets, a growing number of individuals have reported a range of symptoms, including blurred vision, photophobia, dry eyes, tears, and orbital pain, alongside systemic issues like dizziness, headaches, nausea, and emotional disorders [1, 2]. This visual fatigue, characterized by diverse clinical symptoms, is a protracted disease course. The frequent recurrence of visual fatigue significantly impacts the lives and productivity of affected individuals [3]. Moreover, some individuals with visual fatigue also experience emotional distress, including anxiety, depression, and sleep disorders. The gravity of this issue as a substantial public health concern in contemporary society is underscored. Prolonged engagement in close work intensifies the demands on the ocular accommodative system [4], encompassing the ciliary muscle, lens, medial rectus muscle, and pupil. The dysfunction of this system counteracts retinal hyperopia defocusing and results in transient myopia [5]. Spasms of the ciliary muscles and the pupillary sphincter, regulated by the parasympathetic nervous system, can disrupt the balance between the sympathetic and parasympathetic nervous systems, manifesting as physical and mental symptoms. This specific eye condition, identified as accommodative asthenopia (AA) [6], is primarily based on the patient's subjective symptoms and is intertwined with ocular, systemic, and psychological factors.

AA is not considered an independent eye disease. Instead, it is a comprehensive disease interwoven with ocular and systemic factors or psychological elements [7]. Despite this, many ophthalmologists often prioritize the physiological processes of the ocular surface and accommodative mechanisms, inadvertently neglecting the extraocular mechanism of AA [8]. For example, while correcting the refractive system and using artificial tears can partially address accommodative function abnormalities and ocular surface issues, little attention is paid to potential disorders and defects in the high-level visual center. Some studies have indicated that the brain regions involved in processing human ocular accommodative signals are widespread, encompassing the occipital, temporal, marginal, parietal, and frontal lobes, as well as the cerebellum, thalamus, visual networks, cerebellar network, and attention network [9, 10], and so on. However, limited research currently exists on the anatomical structure, metabolism, and function of visual-related brain areas in AA patients. In a previous task-based functional magnetic resonance imaging (fMRI) research [11], it was observed that the activation of some brain areas was increased as the accommodative load increased in patients with AA, notably including the superior parietal gyrus—a key component of the visual attention network. This finding suggested that aberrant brain function plays a role in the pathogenesis of AA.

The glymphatic system [12], a recently discovered waste clearance pathway within the brain, enables to evaluate the efficient fluid transfer between the interstitial space and the perivascular space (PVS). This discovery has significantly contributed to the early diagnosis and treatment of metabolic disorders in the brain, including Parkinson's disease (PD), Alzheimer's disease (AD), and multiple sclerosis [13]. The optic nerve serves as a continuation of the central nervous system, is surrounded by a substantial amount of cerebrospinal fluid (CSF) in the subarachnoid space, and is enveloped by the three layers of meninges: the dura mater, arachnoid mater, and pia mater [14]. Given the revelation of the brain's glymphatic system, it is suggested that the retina and optic nerve might also possess similar clearance pathways. Prior research has highlighted the importance of CSF surrounding the optic nerve in the pathogenesis of glaucoma; it is believed that glaucoma [15], akin to AD, arises from an imbalance between the production and clearance of neurotoxins, including A*β*. Studies proposed that optic disc edema in astronauts could be linked to the dysfunction of the glymphatic system [16]. Despite the limited exploration of this system in the field of AA, uncovering the glymphatic system's existence provides a new perspective for investigating the mechanisms of optic nerve damage in patients with AA.

Diffusion tensor image analysis along the perivascular space (DTI-ALPS) method [17] offered an opportunity to noninvasively explore the glymphatic system in patients with AA. This technique quantifies the movement of water molecules in the slice at the lateral ventricle body level, in the direction of the PVSs, which reflects the activity of the glymphatic system. Importantly, it is a noninvasive method capable of assessing glymphatic function without the need for contrast agent injection [18]. Notably, to the best of our knowledge, no prior studies have employed the DTI-ALPS method to investigate glymphatic system activity in patients with AA. Previous research [19, 20] has demonstrated that individuals with eye diseases exhibit abnormal cortical morphology. In particular, a report highlighted that gray matter (GM) volume mediated the relationship between the glymphatic system and cognition in young-onset AD [21]. Consequently, the compromised glymphatic function observed in patients with AA may be intricately linked to disruptions in cortical morphology.

In this study, we aimed to assess the cortical morphology and glymphatic system activity in patients with AA and investigate the associations between glymphatic system function, cortical morphology, and the severity of patients with AA. We hypothesize that (1) compared with healthy controls (HCs), patients with AA have impaired glymphatic system function and alterations in cortical morphology and that (2) cortical morphology will mediate the effect of glymphatic function on patients with AA.

## 2. Materials and Methods

### 2.1. Participants

One hundred and four subjects were initially recruited from November 2018 to January 2020 from various sources: outpatients from the Ophthalmology Department of Shuguang Hospital Affiliated to Shanghai University of Traditional Chinese Medicine, doctors and graduate students from Shuguang Hospital, and staff members from Jiangsu Runhe Software Co., Ltd. However, seven subjects were excluded from the study due to incomplete magnetic resonance imaging (MRI) scan range (two subjects) and abnormal brain structures (five subjects). Consequently, the MRI data analysis included 47 HCs and 50 patients diagnosed with AA. These participants had no history of brain tumors or head injuries, systemic disease, alcohol/drug dependence, or any other conditions that could potentially affect brain structures and functions, including mental disorders. All participants had attained an education level of junior college or higher, were right-handed, provided informed consent, and voluntarily participated in the study. The study protocol was reviewed and approved by the Ethics Committee of Shuguang Hospital Affiliated to Shanghai University of TCM (Ethics Review No. 2019-749-104-01).

The diagnosis of AA was based on the ocular accommodation parameters [1, 22] and the asthenopia survey scale (ASS) score used to assess the severity of AA. This professional questionnaire was developed by Wenzhou Medical University to assess visual fatigue. The inclusion criteria were as follows: (1) age: 18–60 years old, regardless of gender, ASS score ≥ 16, and astigmatism < −2.00 D, and pseudomyopia allowed; (2) symptoms consistent with the diagnosis of asthenopia [6] ((1) nondurable vision and temporary distant blurred vision; (2) dry eye discomfort with foreign body sensation, burning, itching, photophobia, swelling pain, and tears; and (3) dizziness, headache, anxiety, and insomnia. Asthenopia can be diagnosed if it meets 3/5 of symptoms (1) and (2)); (3) visual function parameter examination showing excessive accommodation—monocular accommodation sensitivity < 6 cycles per minute (cpm) (+ 2.00 D lens difficulty) or combined with accommodation response < +0.25 D, negative relative accommodation < +1.50 D, and binocular accommodation sensitivity (+ 2.00 D lens difficulty) < 3 cpm; and (4) without drug intervention such as antihistamines, anticholinergics, thyroid hormones, antidepressants, antithyroid drugs, and *Ginkgo biloba* extract. The exclusion criteria were as follows: (1) pregnant or lactating women; (2) had major organ dysfunction; (3) had horizontal oblique, vertical oblique, or obvious oblique; (4) suffered from acute conjunctivitis, corneal diseases, other infectious eye diseases, glaucoma, mild to moderate dry eyes with corneal punctate staining, and severe dry eyes; and (5) had a history of eye surgery within 1 month, and patients with visual fatigue had already been treated with eye drops in the previous 1–3 weeks.

### 2.2. MRI Protocols

MRI scans were acquired using a 3 T Siemens MAGNETOM Skyra scanner equipped with a 32-channel head coil. The three-dimensional (3D) magnetization-prepared rapid acquisition gradient echo (MPRAGE) sequence was employed to obtain structural images, and the parameters were as follows: repetition time (TR) = 2200 ms, echo time (TE) = 2.48 ms, flip angle (FA) = 8°, matrix size = 256 × 256, 176 slices, voxel size = 0.9 × 0.9 × 1.0 mm^3^, and field of view (FOV) = 230 mm. The T2W protocol was performed by a turbo spin-echo (TSE) sequence with TR = 4000 ms, TE = 103 ms, FA = 150°, matrix size = 384 × 384, 18 slices, voxel size = 0.6 × 0.6 × 6.0 mm^3^, and FOV = 220 mm. Diffusion tensor imaging (DTI) was performed by spin-echo single-shot echo-planar pulse (EPI) sequence with 64 diffusion-encoding directions, TR/TE = 8500/92 ms, FA = 90°, slice thickness = 2 mm, 58 slices, acquisition matrix = 128 × 128, FOV = 240 mm, and *b*value = 1000 s/mm^2^. Throughout the scanning procedure, participants were instructed to keep their eyes closed, refrain from engaging in cognitive activities, and remain awake.

### 2.3. High-Resolution T1 Volumetric Processing

Voxel-based morphometry (VBM), deformation-based morphometry (DBM), and surface-based morphometry (SBM) analyses were performed in this study using the MATLAB (r2013b) based on the computational anatomy toolbox 12.8.1 (CAT12.8.1, http://www.neuro.uni-jena.de/cat/), which is an extension of SPM12. The preprocessing steps followed the CAT12 standard pipeline with default settings.

#### 2.3.1. VBM Analysis

At first, the images of 3D MPRAGE were manually adjusted to the orientation of the anterior commissure to enhance registration accuracy. Subsequently, a nonlinear deformation field was estimated to achieve the most accurate alignment of the tissue probability maps onto the individual subjects' images. The 3D MPRAGE images were segmented into distinct tissue types, namely, GM, white matter (WM), and CSF. Following this, the segmented data was normalized using the diffeomorphic anatomical registration through the exponentiated Lie algebra toolbox (DARTEL) to the standard Montreal Neurological Institute (MNI) space. The isotropic voxel resolution employed was 1.5 × 1.5 × 1.5 mm^3^ [23]. Finally, to facilitate the statistical analysis, we applied a Gaussian filter with a full width at half maximum (FWHM) of 8 mm. Subsequently, the total intracranial volume (TIV) was estimated for each individual by summing GM, WM, and CSF compartments [24].

#### 2.3.2. SBM Analysis

The parameters used for cortical thickness estimation involved a projection-based approach, by quantifying the distance between the inner and outer cortical surfaces. These surfaces demarcated the boundaries between WM and GM, as well as between GM and CSF. Additionally, three other geometric measures were derived: sulcal depth, cortical complexity, and degree of gyrification. Sulcal depth represents the depth of grooves or fissures on the brain surface and reflects the folding pattern of the cerebral cortex. The complexity of the cortical folding pattern was quantified by cortical complexity, which was determined by the fractal dimension. The degree of gyrification, which measures the cerebral cortex's complexity and folding, is computed using the mean surface curvature of the brain in the manner described as follows [25]:

 $$\begin{array}{c} \text{Gyrification index}=\frac{\text{area of local pial surface}}{\text{area of local hull surface}} \end{array}$$

Each hemisphere underwent individual surface reconstruction using the projection-based thickness method. This method corrected surface mesh defects and accounted for spherical inflation and registration, which could ensure accurate topology. For facilitating intersubject analysis, a modified DARTEL algorithm was utilized during spherical registration to project the matrix onto a standardized spherical surface. The merging of meshes from the right and left hemispheres resulted in a unified mesh. Following this, smoothing of the cortical thickness images was accomplished by a 15-mm FWHM Gaussian kernel, with other parameters smoothed employing a 20-mm FWHM Gaussian kernel.

#### 2.3.3. DBM Analysis

For each participant, 3D MPRAGE images were transformed into a DBM map using a previously described procedure [26]. The nonlinear transformation obtained from the previous VBM analysis was inverted to derive deformation fields that mapped voxel coordinates (*χ*1, *χ*2, and *χ*3) in the subject's native space to corresponding voxels in the MNI template (*u*1(*χ*), *u*2(*χ*), and *u*3(*χ*)). Then, using a first-order approximation, Jacobian matrices of the deformation were generated and estimated. The voxel-wise relative deformation value, represented as (|*J*| − 1), was calculated to create the DBM maps. This value indicated the deformation (positive for expansion and negative for shrinkage) of each brain voxel during registration to the MNI template. The resulting data was then resampled to a voxel size of 1.5 × 1.5 × 1.5 mm^3^. Finally, a Gaussian filter with an 8-mm FWHM was applied to facilitate statistical analysis.

### 2.4. DTI-ALPS Index Calculation

**Figure 1:**
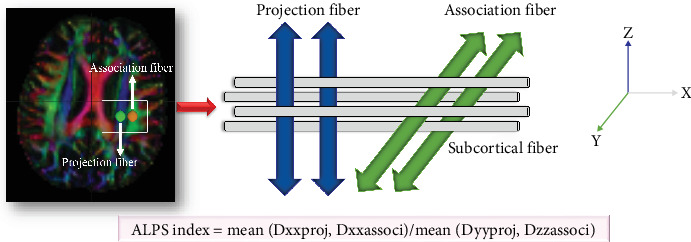
Method for acquiring the diffusion tensor image analysis along the perivascular space (DTI-ALPS) index in this study. Dxxproj, diffusivity along the *X*-axis in the projection fiber; Dxxassoci, diffusivity along the *X*-axis in the association fiber; Dyyproj, diffusivity along the *Y*-axis in the projection fiber; Dzzassoci, the diffusivity along the *Z*-axis in the association fiber.

We used DSI Studio graphic-user interface software Version 10.15 (DSI Studio GUI; https://dsi-studio.labsolver.org/) for preprocessing the diffusion tensor images. The program includes the following [27, 28]: (1) phase distortion in DTI images was corrected using reverse phase encoding (PE) volumes, and this process was followed by further correction for eddy current and head movement artifacts; (2) generation of a color-coded fractional anisotropy map and diffusive maps for the principal diffusion directions (*x*-axis: right-left or Dxx, *y*-axis: anterior-posterior or Dyy, and *z*-axis: inferior-superior or Dzz) was performed; and (3) both linear and nonlinear transformations were applied to translate the reconstructed image, also known as fractional anisotropy map, into the template space. The resulting transformation matrix was then utilized to align all the diffusive maps. Subsequently, circular regions of interest (ROIs) (5 mm × 5 mm) were placed at the lateral ventricle body level in projection fiber and association fiber regions. The fiber orientation and diffusivities in three spatial directions (*x*-, *y*-, and *z*-axes) were extracted at the voxel level within the ROIs (Figure 1). Finally, the DTI-ALPS index was calculated as the ratio between the mean diffusivity (MD) in the area of projection fibers (Dxxproj) and association fibers (Dxxassoci) on the *x*-axis and that of the projection fibers (Dyyproj) on the *y*-axis and the association fibers (Dzzassoci) on the *z*-axis as follows [17]:

 $$\begin{array}{c} \text{DTI}­\text{ALPS index}=\frac{\text{mean}\left(\text{Dxxproj},\text{Dxxassoci}\right)}{\text{mean}\left(\text{Dyyproj},\text{Dzzassoci}\right)} \end{array}$$

Two neuroradiologists (5 and 8 years of experience, respectively) independently placed the ROIs. We assessed the overall glymphatic function of the participants using the average bilaterally calculated analysis along the perivascular space (ALPS) index. For each participant, a neuroradiologist independently placed the ROIs and calculated the DTI-ALPS index independently. The results measured by the two neuroradiologists were then averaged as the final result for each subject.

### 2.5. Statistical Analysis

The two-sample *t*-test was employed to compare age, education years, ASS scores, and equivalent spherical lens values between patients with AA and HCs. Pearson's chi-square test was utilized to examine gender differences across groups. All these statistical procedures were conducted using IBM SPSS Statistics software (Version 25). Statistical significance was determined by a *p* value less than 0.05. The two-sample *t*-test, accounting for age, gender, and education years as covariates, was utilized to contrast the difference in global structural morphometry between patients with AA and HCs. Statistical significance was defined as a *p* value less than 0.05. Partial correlation analyses were performed to investigate the relationship between global structural morphometry and clinical parameters (disease duration, ASS, and visual function parameter) after adjusting age, sex, and education years. The Bonferroni correction method was selected to correct the multiple comparisons with the significant threshold set at *p* < 0.05/*N*, where *N* represented the times of comparisons.

The statistical analyses of cortical thickness, sulcal depth, cortical complexity, gyrification index, and GM volume (by DBM and VBM) between-group differences were assessed with two-sample *t*-tests with age, gender, and education years as covariates. In addition, TIV was entered as a covariate for differences in VBM models. The false discovery rate (FDR) was selected for multiple comparison correction, which first set the voxel level threshold to *p* < 0.001 and then set the cluster level to *p* < 0.05. Partial correlation analyses were performed to explore the associations between cortical thickness/volume, gyrification, sulcal depth, cortical complexity, gyrification index, and clinical parameters (disease duration, ASS, and visual function parameter) after adjusting age, sex, and education years. *p* < 0.05 was considered statistically significant, and the Bonferroni correction method was used for multiple comparison corrections.

The comparison of the diffusivities and ALPS index between patients with AA and HCs employed the two-sample *t*-test. The age, gender, and education years were used as covariates, and *p* < 0.05 was considered statistically significant. Multiple corrections were applied when we performed statistical analysis for diffusivities (Bonferroni correction, *p* = 0.0028[0.05/18]). Interobserver agreement for DTI-ALPS was evaluated using intraclass correlation coefficients (ICCs) across all participants (range and correlation: 0.00–0.20, poor; 0.21–0.40, fair; 0.41–0.60, moderate; 0.61–0.80, good; and 0.81–1.00, excellent).

Building upon the significant findings from the above statistical analyses, we performed a mediation analysis to investigate whether the identified cortical morphology features mediated the association between them and the ALPS index and ASS scores. In this analysis, the ALPS index served as the independent variable, ASS scores as the dependent variable, and alterations in cortical morphology as the mediator variable, with age, sex, and education years included as covariates. Consistent with previous research methodologies [29], the mediation model was calculated in SPSS 25 using PROCESS v3.4. This involved the application of Model 4 (simple mediation model) from the macro created by Andrew Hayes (http://processmacro.org/). The mediation effect was deemed significant if the 95% confidence interval (CI) for the indirect effect excluded zero.

## 3. Results

### 3.1. Demographic and Clinical Characteristics

Table 1 presents demographic and clinical characteristics of the 50 patients and 47 HCs. Briefly, the two groups did not differ in age (*t* = 2.68, *p* = 0.075), gender (*χ*^2^ = 0.457, *p* = 0.499), education years (*t* = −1.39, *p* = 0.441), or equivalent spherical lens (R: *t* = −0.30, *p* = 0.922; L: *t* = 0.71, *p* = 0.872). There was a significant group difference in the ASS score. As shown, patients had higher ASS scores (*t* = 8.86, *p* = 0.002) than HCs.

### 3.2. Global Structural Morphometry Alterations in Patients With AA

**Figure 2:**
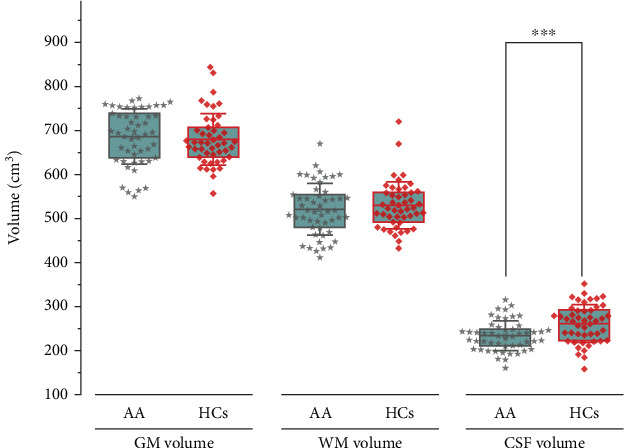
Comparison of global structural morphometry between patients with AA and HCs. AA, accommodative asthenopia; HCs, healthy controls; GM, gray matter; WM, white matter; CSF, cerebrospinal fluid. ⁣^∗∗∗^*p* < 0.001.

Figure 2 shows that there were no significant differences between patients with AA and HCs in global GM and WM volume when adjusting TIV, age, gender, and education years. A significant reduction in global CSF volume was observed in patients with AA compared to HCs (*p* < 0.001). However, there was no significant correlation between global CSF volume and clinical indicators after controlling for age, gender, and education years (*p* > 0.05).

### 3.3. Alterations of Cortical Morphology in Patients With AA

**Figure 3:**
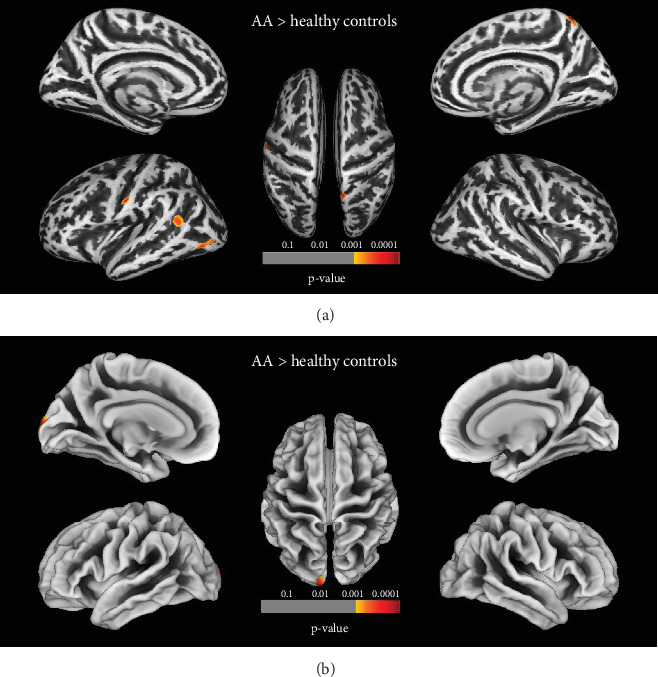
Comparison of the cortical morphology of patients with AA and HCs. (a) Sulcal depth: the warm color regions representing significantly deeper sulcal depth in patients with AA (FDR, voxels *p* < 0.001, clusters *p* < 0.05). (b) Cortical thickness: the warm color regions representing significantly thicker cortical regions in patients with AA (FDR, voxels *p* < 0.001, clusters *p* < 0.05). The color bar indicates the voxel-wise *t* value. AA, accommodative asthenopia; HCs, healthy controls.

**Figure 4:**
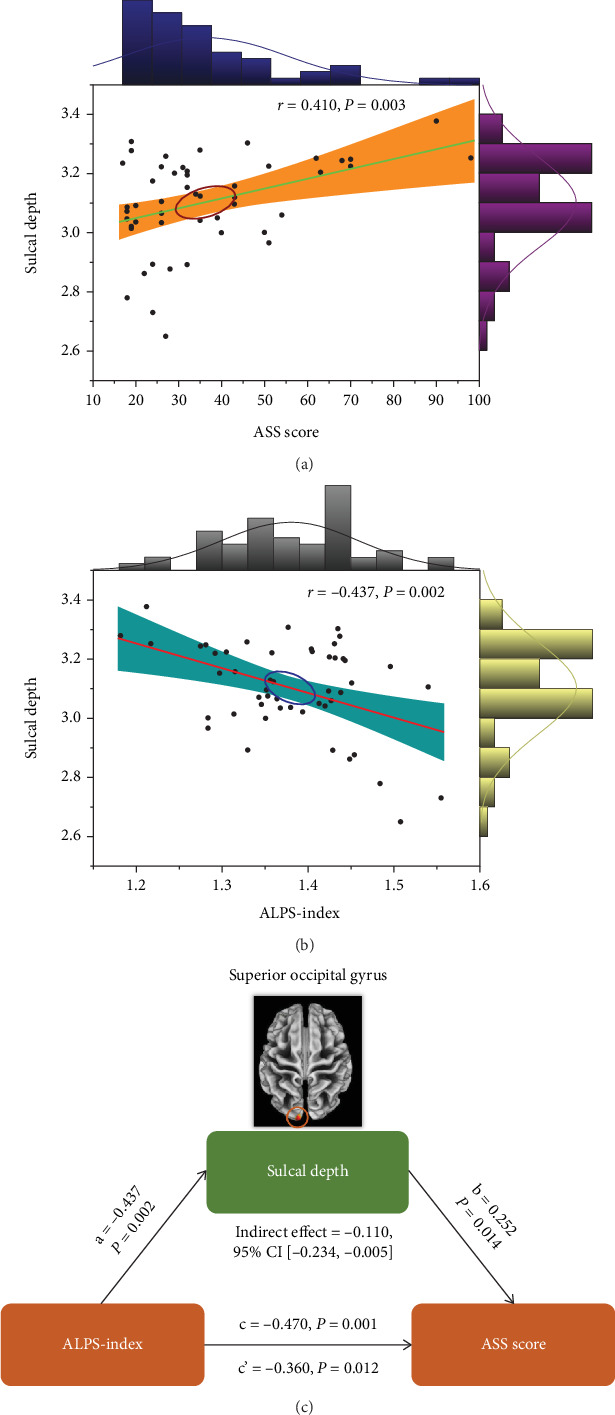
The relationship between sulcal depth of the SOG.L, ALPS index, and ASS score. (a) Correlation analysis between sulcal depth of the SOG.L and ASS score. (b) Correlation analysis between sulcal depth of the SOG.L and ALPS index. (c) Mediation analysis between the ALPS index (*X*) and ASS score (*Y*), with sulcal depth of the SOG.L as the mediator (*M*). ALPS, analysis along the perivascular space; ASS, asthenopia survey scale; SOG.L, left superior occipital gyrus.

**Figure 5:**
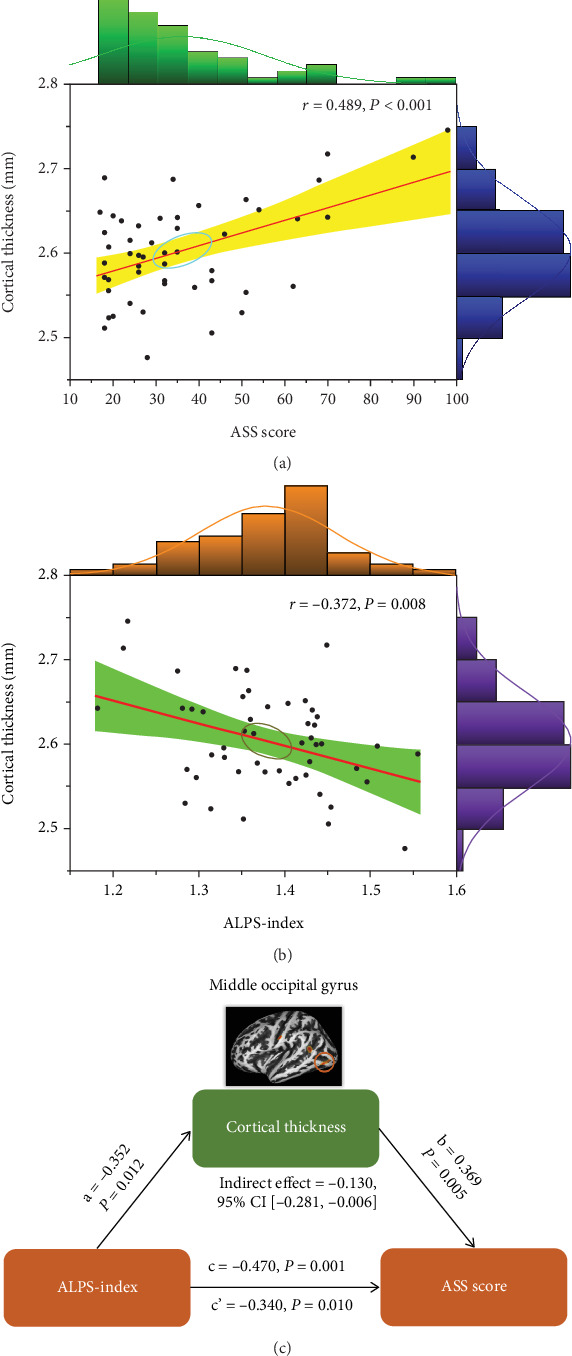
The relationship between cortical thickness of the MOG.L, ALPS index, and ASS score. (a) Correlation analysis between cortical thickness of the MOG.L and ASS score. (b) Correlation analysis between cortical thickness of the MOG.L and ALPS index. (c) Mediation analysis between the ALPS index (*X*) and ASS score (*Y*), with cortical thickness of the MOG.L as the mediator (*M*). ALPS, analysis along the perivascular space; ASS, asthenopia survey scale; MOG.L, left middle occipital gyrus.

Compared to HCs, patients with AA showed significantly increased sulcal depth in cluster located in the left superior occipital gyrus (SOG.L) (*p* < 0.05, FDR corrected). Patients with AA had increased cortical thickness in four clusters compared to HCs, including the left superior temporal gyrus (STG.L), left middle occipital gyrus (MOG.L), left postcentral gyrus (PoCG.L), and left precuneus (PCUN.L) (*p* < 0.05, FDR corrected) (Figures 3(a) and 3(b) and Table 2). No clusters of reduced sulcal depth and cortical thickness were observed in patients with AA relative to HCs. In addition, no significant differences were found in the whole-brain VBM and DBM analyses between patients with AA and HCs (*p* > 0.05, FDR corrected). The correlation analysis results are presented in Figures 4(a) and 5(a). The sulcal depth of the SOG.L was significantly positively correlated with the ASS score in patients with AA (*r* = 0.410, *p* = 0.003), and a positive correlation was found between the cortical thickness of the MOG.L and ASS score (*r* = 0.489, *p* < 0.001). There was no significant correlation between other abnormal cortical morphology regions and clinical data.

### 3.4. Differences in the Diffusivities and ALPS Index Between Patients With AA and HCs

**Figure 6:**
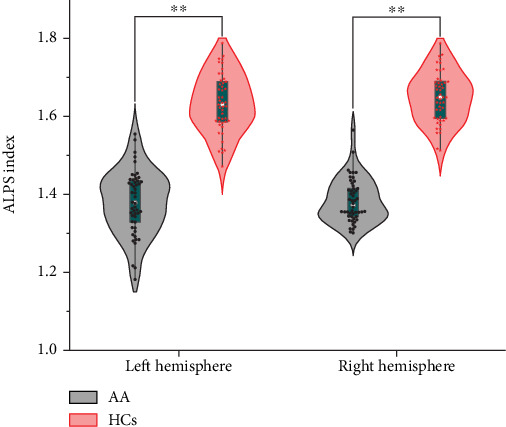
The differences of the left ALPS index and right ALPS index between patients with AA and HCs. ⁣^∗∗^*p* < 0.01. AA, accommodative asthenopia; HCs, healthy controls.

The diffusivity along the *y*-axis of the projection fiber was higher and along the *y*-axis of the association fiber was lower in patients with AA compared to HCs (Table 3). For both the left ALPS index (ICC = 0.916, 95% CI 0.88–0.94, *p* < 0.001) and the right ALPS index (ICC = 0.905, 95% CI 0.86–0.93, *p* < 0.001) (Figures S1 and S2), the interobserver reliability for the ALPS index acquired from the two neuroradiologists was excellent. The bilateral ALPS index of patients with AA was lower than that of HCs (*p* = 0.002 and 0.005, respectively) (Figure 6). Subsequently, in patients with AA, the ALPS index was negatively associated with sulcal depth of the SOG.L (*r* = −0.437, *p* = 0.002) and cortical thickness of the MOG.L (*r* = −0.372, *p* = 0.008) (Figures 4(b) and 5(b)). There was no significant relationship between the ALPS index and the other abnormal cortical morphology regions in patients with AA.

### 3.5. Associations Among ALPS Index, Cortical Morphology, and ASS Score

A mediation analysis was conducted on patients with AA to investigate the relationship among the ALPS index, cortical morphology, and ASS score. The ALPS index was designated as the independent variable (*X*), the ASS score as the dependent variable (*Y*), and alterations of cortical morphology as a mediating variable (*M*). The mediation analysis revealed that sulcal depth of the SOG.L partly mediated the relationship between the ALPS index and ASS score (indirect effect = −0.110, 95% CI [−0.234, −0.005]) with 5000 bootstrap samples after controlling for age, sex, and education years (Figure 4(c)). Meanwhile, it was found that the cortical thickness of the MOG.L significantly partly mediated the relationship between the ALPS index and ASS score (indirect effect = −0.130; 95% CI [−0.281, −0.006]) (Figure 5(c)). The mediation effect accounted for 23.40% and 27.66% of the total effects for the relationship between the ALPS index and ASS score, respectively.

## 4. Discussion

In this study, we primarily investigated the alterations in the glymphatic system function and cortical morphology in patients with AA and further explored their relationship with the severity of AA. Our study revealed that patients with AA exhibited impaired glymphatic function and abnormal morphological changes in certain areas of the visual cortex, and the alterations in cortical morphology partially mediated the impact of the DTI-ALPS index on the severity of AA.

The human eye's regulation is facilitated by a functional unit that includes the ciliary muscle, suspensory ligament, and lens. Regulatory stimuli can excite neurons in the brain regions related to regulatory functions. Thereby, nerve impulses are released to control the contraction of the ciliary muscle. This will make the suspensory ligament relax, which changes the curvature level of the lens and keeps them with a good elasticity coefficient. So the objects at close distance can be quickly and clearly imaged on the retina [5]. The popularity of computers and mobile terminals has led to excessive regulatory loads caused by near-distance work stimuli, which is the main reason for most visual fatigue patients [4]. The main method for treating excessive regulation is to apply eye drops that have a paralyzing effect on the ciliary muscles, but it can only partially relieve the tension of the ciliary muscles. The fundamental reason for this is that there is abnormal excitement in the functional area of the brain that controls the function of eye regulation. Numerous brain regions have been reported to control near reflexes, including the occipital, parietal, frontal, and temporal lobes and cerebellum [9, 10]. However, no literature has reported that the cortical functional area of the brain controlled the regulation function of visual fatigue. The results of this study's brain morphology research showed that the sulcus depth of SOG.L was significantly increased, a region linked to visual processing. This suggested a potential adaptation or response to visual strain in patients with AA. A prior study [11] reported that the increased activation in the occipital cortex during accommodative tasks in individuals with AA indicated a possible compensatory mechanism to maintain visual performance. The increased sulcal depth in SOG.L might reflect structural changes associated with this functional adaptation. Furthermore, the cortical thickness of the STG.L, MOG.L, PoCG.L, and PCUN.L was increased. The involvement of these regions suggested a broader cortical restructuring in AA, which incorporated areas associated with sensory integration and higher-order visual processing [30, 31]. The absence of clusters with reduced sulcal depth or cortical thickness in AA patients compared to HCs may be partly attributed to variability within our study population, leading to these differences. Our cohort may differ from those in earlier studies in terms of clinical characteristics such as disease duration, severity, or age. These factors could influence the nature and extent of neuroanatomical changes, potentially resulting in augmentation in certain regions rather than reduction. Another possibility is that the structural brain changes observed in our cohort reflect compensatory neuroplastic processes. In response to underlying neurodegenerative pathology, some regions may undergo structural augmentation as a compensatory mechanism. Further findings revealed that the sulcus depth of the superior occipital gyrus and the cortical thickness of the middle occipital gyrus were positively correlated with the ASS score. These results showed that the superior occipital gyrus and the middle occipital gyrus were involved in the neural circuit of eye regulation function from the perspective of brain tissue morphology. The positive correlation observed in our study indicates that as ASS scores increase, there is a corresponding increase in sulcal depth and cortical thickness in these regions. However, it does not necessarily imply a straightforward pathological worsening of AA symptoms, and this finding might reflect neuroadaptive or compensatory changes in response to prolonged visual stress, which could influence cortical structure.

In vivo, the flow path of CSF was usually shown by fluorescent tracing with a two-photon imaging technology. It demonstrated that CSF entered the brain parenchyma through the PVS surrounding arteries, exchanged with brain interstitial fluid, and was then cleared along the venous side space. This confirmed the existence of a glymphatic-like clearance system in brain tissue similar to other organs [32]. The functional composition of the glymphatic system mainly includes three parts: the inflow of CSF in the arterial side space, the fluid exchange in the brain parenchyma and the removal of metabolic waste, and the outflow of tissue fluid in the venous side space [33]. The glymphatic system is crucial for the removal of metabolic products in the body and the maintenance of body fluid homeostasis. Previous research posited a deficiency of glymphatic circulation in the eye, with metabolic byproducts primarily expelled via the aqueous humor circulation focused in the eye's anterior segment. However, this theory fell short in elucidating the efficient removal of neurotoxic substances generated by the highly metabolic retinal neurons [34]. Wang et al. [35] proposed the notion of the eye glial glymphatic system. In this system, the aqueous humor is combined with the retinal interstitial fluid at the rear of the eye to form a mixed fluid. This fluid, driven by the Water Channel Protein 4 at the neuroglial cell terminus, traversed the sieve plate to reach the PVS of the retinal vein. Ultimately, it is absorbed by the dural glymphatic vessels and enters the glymphatic circulation. This newfound understanding of the glymphatic system opened up a fresh avenue for exploring the pathogenesis of ocular disorders such as glaucoma [36], aging [37], and optic disc edema [16]. Extended periods of close-range tasks and stress in patients with AA resulted in overaccommodation, which triggered an excessive stimulation of the parasympathetic nervous system. This, in turn, led to an accumulation of local metabolic byproducts and kept the ciliary muscle in a state of persistent contraction, thereby inhibiting its ability to relax [38]. CSF constitutes a primary component of the extracellular fluid within the central nervous system and plays a pivotal role in waste clearance in the rodent brain [39]. In this study, the CSF volume of patients with AA was significantly reduced compared with healthy subjects, which might be related to their slow drainage, indicating a dysfunction of the glymphatic system circulation. In this study, patients with AA exhibited impaired glymphatic system functionality which implied a diminished clearance efficiency of the glymphatic system and the spasms of the ciliary muscle. The DTI-ALPS technique utilized in this study could only reflect the transient functionality of the glymphatic system during MRI. Future research should focus on elucidating the operational dynamics of the glymphatic system. Overall, the ALPS index, a novel biomarker reflecting glymphatic system function, may contribute to exploring the neurophysiological underpinnings of AA. Incorporating the ALPS index into clinical evaluations could provide an objective and quantitative measure of underlying glymphatic disturbances, complementing traditional methods that rely on subjective symptom reporting. This advancement may allow clinicians to better assess the severity of AA and identify subtle, neurophysiological changes that precede more noticeable symptoms. Moreover, the ALPS index could play a critical role in monitoring disease progression or the response to treatment in AA patients. By tracking changes in glymphatic function over time, clinicians may gain insights into the effectiveness of various therapeutic strategies. By targeting the glymphatic system, clinicians may be able to reduce the neurophysiological strain associated with AA, offering novel approaches to symptom management and improving overall patient outcomes.

Our study further identified a negative correlation between the ALPS index and both the sulcal depth of the SOG.L and the cortical thickness of the MOG.L. These morphological changes in the visual cortex might be associated with the activation of the cortical functional area supporting ocular accommodation. The ALPS index reflected the speed of glymphatic circulation. This correlation suggested that the functional area of the ocular accommodation cortex in AA patients can influence their glymphatic system functionality, leading to impediments in waste expulsion. Furthermore, the mediation analysis among cortical morphological changes, the ALPS index, and the severity of asthenopia showed that the sulcal depth of the SOG.L and the cortical thickness of the MOG.L significantly mediated the relationship between the ALPS index and the severity of asthenopia. Previous studies [40] have indicated the involvement of the brain's glymphatic system in the pathological processes and recuperation of cerebral functions in neurological disorders, including stroke, AD, and traumatic brain injuries. Our findings suggested that the brain's glymphatic system also plays a role in the process of accommodation dysfunction in AA. Moreover, alterations in cortical morphology were pivotal in this process. Therefore, future research should focus on the role of cortical morphology within the circulation of the brain's glymphatic system.

### 4.1. Limitations

There were several limitations in this study. First, the absence of a control group with other visual or attention-related disorders and the relatively small sample size potentially limited the generalizability of our findings. To enhance the validity and robustness of our results, future studies should involve a larger sample size and more diverse disorders. Second, our research employed a cross-sectional design, which did not allow for examining the temporal dynamics of the neuroanatomical and glymphatic function changes in AA. A longitudinal study design would be more helpful for investigating the evolution or resolution of these changes over time and the correlation with clinical outcomes. At last, our research primarily focused on the structural and glymphatic function aspects of the brain, neglecting to assess the functional implications of these changes. Future studies aimed at understanding the neural activity and connectivity patterns associated with AA should incorporate functional neuroimaging techniques, such as functional MRI or electroencephalography.

## 5. Conclusion

In conclusion, we found that patients with AA had impaired glymphatic system function and alterations in cortical morphology. Furthermore, cortical morphology was a significant mediator in the relationship between the glymphatic system function and the severity of asthenopia in patients with AA. Our findings contributed to the evolving understanding of the underlying pathophysiological mechanisms of AA.

## Figures and Tables

**Table 1:** Demographic and clinical characteristics.

**Characteristics**	**AA (** **n** = 50**)**	**HCs (** **n** = 47**)**	**p** ** value**
Age (years)	31.48 ± 5.50	28.36 ± 4.34	0.075
Gender (female/male)	30/20	25/22	0.499
Education (years)	16.95 ± 1.88	17.55 ± 1.80	0.441
Disease duration (years)	3.58 ± 2.34	—	
ASS score	39.18 ± 18.94	8.17 ± 6.55	0.002
Equivalent spherical lens (R, diopter)	−3.37 ± 2.68	−3.15 ± 2.64	0.922
Equivalent spherical lens (L, diopter)	−3.23 ± 2.59	−2.95 ± 2.68	0.872

Abbreviations: AA, accommodative asthenopia; ASS, asthenopia survey scale; HCs, healthy controls.

**Table 2:** Clusters showing significantly changed cortical morphology in patients with AA compared to HCs.

**Measurement**	**Brain region**	**Hemisphere**	**Peak MNI coordinates**			**Cluster size (voxels)**	**Peak intensity**
			**X**	**Y**	**Z**		
SBM							
Sulcal depth	Superior occipital gyrus	L	−18	−89	26	259	3.911
Cortical thickness	Superior temporal gyrus	L	−40	16	−23	233	5.005
	Middle occipital gyrus	L	−35	−85	14	329	4.234
	Postcentral gyrus	L	−45	−25	49	153	3.456
	Precuneus	R	12	−58	45	175	4.023

Abbreviations: AA, accommodative asthenopia; HCs, healthy controls; SBM, surface-based morphometry.

**Table 3:** Differences between the diffusivities along the fiber axes and DTI-ALPS index in patients with accommodative asthenopia and healthy controls.

	**AA (** **n** = 50**)**	**HCs (** **n** = 47**)**	**p** ** value**
Right projection fiber			
Dxx (× 10^−3^ mm^2^/s)	0.589 ± 0.081	0.608 ± 0.074	0.407
Dyy (× 10^−3^ mm^2^/s)	0.499 ± 0.124	0.372 ± 0.086	0.001
Dzz (× 10^−3^ mm^2^/s)	1.334 ± 0.127	1.064 ± 0.137	0.023
Right association fiber			
Dxx (× 10^−3^ mm^2^/s)	0.595 ± 0.094	0.615 ± 0.127	0.325
Dyy (× 10^−3^ mm^2^/s)	1.106 ± 0.121	1.365 ± 0.123	0.001
Dzz (× 10^−3^ mm^2^/s)	0.341 ± 0.094	0.328 ± 0.084	0.367
Right subcortical fiber			
Dxx (× 10^−3^ mm^2^/s)	1.071 ± 0.155	1.152 ± 0.108	0.528
Dyy (× 10^−3^ mm^2^/s)	0.604 ± 0.186	0.655 ± 0.167	0.487
Dzz (× 10^−3^ mm^2^/s)	0.571 ± 0.184	0.628 ± 0.195	0.249
DTI-ALPS_R_ index	1.383 ± 0.056	1.649 ± 0.065	0.002
Left projection fiber			
Dxx (× 10^−3^ mm^2^/s)	0.594 ± 0.074	0.596 ± 0.071	0.746
Dyy (× 10^−3^ mm^2^/s)	0.481 ± 0.131	0.381 ± 0.074	0.006
Dzz (× 10^−3^ mm^2^/s)	1.115 ± 0.125	1.104 ± 0.129	0.534
Left association fiber			
Dxx (× 10^−3^ mm^2^/s)	0.546 ± 0.088	0.638 ± 0.122	0.014
Dyy (× 10^−3^ mm^2^/s)	1.101 ± 0.076	1.281 ± 0.118	0.009
Dzz (× 10^−3^ mm^2^/s)	0.351 ± 0.101	0.339 ± 0.093	0.338
Left subcortical fiber			
Dxx (× 10^−3^ mm^2^/s)	1.051 ± 0.127	1.122 ± 0.124	0.624
Dyy (× 10^−3^ mm^2^/s)	0.646 ± 0.174	0.656 ± 0.168	0.714
Dzz (× 10^−3^ mm^2^/s)	0.617 ± 0.067	0.629 ± 0.175	0.515
DTI-ALPS_L_ index	1.379 ± 0.081	1.633 ± 0.075	0.005

Abbreviations: AA, accommodative asthenopia; DTI-ALPS_L_, left-hemispheric diffusion tensor image analysis along the perivascular space; DTI-ALPS_R_, right-hemispheric diffusion tensor image analysis along the perivascular space; HCs, healthy controls.

## Data Availability

Medical images used to support the results of this study are not yet available due to patient confidentiality. Statistical data used to support the results of this study are available from the corresponding authors upon request.

## Supporting Information

Supporting Information Additional supporting information can be found online in the Supporting Information section. A supporting information file is associated with this manuscript. This file contains the following items, all referenced in the main text. Figure S1: Bland–Altman plots of the interobserver reliability for the ALPS index in the left hemisphere acquired from the two radiologists. Figure S2: Bland–Altman plots of the interobserver reliability for the ALPS index in the right hemisphere acquired from the two radiologists.
