# Supplementary material for: Cortical Morphology Alterations Mediate the Relationship Between Glymphatic System Function and the Severity of Asthenopia
Source: Int J Biomed Imaging. 2025 Feb 25;2025:4464776. doi: 10.1155/ijbi/4464776 (PMC11879604; doi:10.1155/ijbi/4464776)
Supplement: Supporting Information — Additional supporting information can be found online in the Supporting Information section. A supporting information file is associated with this manuscript. This file contains the following items, all referenced in the main text. Figure S1: Bland–Altman plots of the interobserver reliability for the ALPS index in the left hemisphere acquired from the two radiologists. Figure S2: Bland–Altman plots of the interobserver reliability for the ALPS index in the right hemisphere acquired from the two radiologists. [file 4464776.f1.docx]

**Supplementary Fig. S1** Bland-Altman plots of the interobserver reliability for the ALPS-index in the left hemisphere acquired from the two radiologists.





ICC, intraclass correlation coefficient.

**Supplementary Fig. S2** Bland-Altman plots of the interobserver reliability for the ALPS-index in the right hemisphere acquired from the two radiologists.





ICC, intraclass correlation coefficient.
